# Age-Dependent Activation and Neuronal Differentiation of Lgr5+ Basal Cells in Injured Olfactory Epithelium *via* Notch Signaling Pathway

**DOI:** 10.3389/fnagi.2020.602688

**Published:** 2020-12-17

**Authors:** Xuewen Li, Meimei Tong, Li Wang, Yumei Qin, Hongmeng Yu, Yiqun Yu

**Affiliations:** ^1^School of Life Sciences, Shanghai University, Shanghai, China; ^2^Department of Otolaryngology, Eye, Ear, Nose and Throat Hospital, Shanghai Key Clinical Disciplines of Otorhinolaryngology, Fudan University, Shanghai, China; ^3^School of Food Science and Bioengineering, Zhejiang Gongshang University, Hangzhou, China; ^4^Research Units of New Technologies of Endoscopic Surgery in Skull Base Tumor, Chinese Academy of Medical Sciences, Beijing, China; ^5^Ear, Nose and Throat Department, Yuecheng People’s Hospital, Shaoxing, China

**Keywords:** olfactory epithelium, olfactory sensory neuron, aging, LGR5, Notch, organoid

## Abstract

Aging is an important factor affecting function of smell, leading to the degeneration of mature olfactory sensory neurons and inducing the occurrence of smell loss. The mammalian olfactory epithelium (OE) can regenerate when subjected to chemical assaults. However, this capacity is not limitless. Inactivation of globose basal cells and failure to generate sensory neurons are the main obstacles to prevent the OE regeneration. Here, we found the significant attenuation in mature sensory neuronal generation and apparent transcriptional alternation in the OE from aged mice compared with young ones. The recruitment of leucine-rich repeat-containing G-protein coupled receptor 5 (Lgr5)-positive cells in injured OE was weakened in aged mice, and more Lgr5+ cells remained quiescence in aged OE postinjury. Lineage-traced progenies from Lgr5+ cells were significantly fewer in the OE with aging. Moreover, Notch activation enhanced the neuronal regeneration in aged OE, making the regenerative capacity of aged OE comparable with that of young animals after injury. The growth and morphology of three-dimensional (3D)-cultured organoids from the OE of young and aged mice varied and was modulated by small molecules regulating the Notch signaling pathway. Thus, we concluded that activation of Lgr5+ cells in injured OE was age dependent and Notch activation could enhance the capacity of neuronal generation from Lgr5+ cells in aged OE after injury.

## Introduction

The mammalian olfactory epithelium (OE) is a neuroepithelial structure that has self-renewal capacity throughout life. Horizontal basal cells (HBCs) and globose basal cells (GBCs) are two types of stem/progenitor cell populations responsible for the regeneration of injured OE (Jang et al., 2003; Chen et al., 2004; Schnittke et al., 2015; Schwob et al., 2017). When subjected to chemical assaults, dormant stem cells (mainly HBCs) are recruited to generate GBCs and then differentiate into olfactory sensory neurons, sustentacular cells, and other cellular subtypes constituting the OE (Leung et al., 2007). Acute injury drives proliferating cells to differentiate into immature neurons expressing Tuj1 and GAP43, and then grow into mature neurons expressing olfactory marker protein (OMP) and PGP9.5. However, the neuro-regenerative capacity of basal cells in the OE is not limitless and aging is a principal element causing the sensory neuronal death and inactivation of GBCs (Child et al., 2018). Studies on both human and laboratory animals such as mice have shown age-related morphology and functional changes in the olfactory nerve epithelium (Mobley et al., 2014). Age-associated decline in the neuro-regenerative capacity of the injured OE is related with the weakness in proliferative activity (Suzukawa et al., 2011). Spontaneous lesions occur in the neuroepithelium and Bowman’s glands in mouse olfactory mucosa with progression of aging (Kondo et al., 2009). Several biomarkers have been identified as hallmarks of the aged olfactory mucosa, such as low expression of extracellular matrix genes (Ueha et al., 2018b) and ApoE deficiency (Zhang et al., 2018). With the increase of age, the recovery rate slows down and respiratory metaplasia may occur (Suzukawa et al., 2011; Child et al., 2018). Strategies against age-related neuronal degradation have been reported, including insulin-like growth factor 1 (IGF-1) administration (Ueha et al., 2018a), intranasal treatment with fibroblast growth factor-2 (FGF-2; Fukuda et al., 2018), activation of inositol trisphosphate receptor type 3 (IP3R3), and the neuroproliferative factor neuropeptide Y (NPY) signaling (Jia and Hegg, 2015).

The Notch signaling pathway plays important roles in the entire organism development process and in maintaining tissue self-renewal (Artavanis-Tsakonas and Muskavitch, 2010). Activation of Notch signaling transduction leads to proteolysis of the Notch receptor, allowing intracellular domain of Notch (NICD) to enter the nucleus, and subsequently binds to DNA-binding proteins and assembles transcription complexes that activate corresponding downstream target genes (Kopan and Ilagan, 2009). Notch expression is present in the neurogenesis of the peripheral olfactory system (Doi et al., 2004), and various Notch subtypes play different roles in determining neuronal and glial lineage during development of the OE (Carson et al., 2006). Notch signaling is necessary to direct the neuronal differentiation in the OE regeneration (Herrick et al., 2018), and Notch1 is required to maintain dormancy of reserve HBCs (Herrick et al., 2017). Our previous work indicated that Notch1 activation enhanced the proliferation in leucine-rich repeat-containing G-protein coupled receptor 5 (Lgr5)-positive progenitor cells and regulated the generation of mature sensory neurons in the OE (Dai et al., 2018). Thus, it is of interest to elucidate whether the effect of aging on the OE regeneration will be counteracted *via* Notch signaling activation.

In this study, we elucidated the transcriptional change in the OE from young and aged mice. Aging attenuated the recruitment of Lgr5+ cells in injured OE. Previous work reported that Lgr5 marked GBCs in the OE (Chen et al., 2014) and Lgr5+/Notch1+ cells participated in the OE regeneration postinjury (Dai et al., 2018). Based on these findings, here, we showed that aging altered the stemness of Lgr5+ cells in injured OE, keeping more Lgr5+ cells in dormancy. In aged OE, the capacity of neuronal generation from Lgr5+ cells were weakened, while Notch activation mimicking by NICD overexpression offset aging-induced intervention on neuronal regeneration in injured OE. *In vitro*-cultured OE organoids from aged mice showed more apparent cystic morphology compared with those from young mice, which was altered by chemical treatment regulating the Notch signaling. Thus, these data supported the conclusion that aging was a critical factor determining the participation of Lgr5+ cells in OE regeneration, and the adverse effect of aging on neuronal differentiation was counteracted by Notch activation. This study supplemented new evidence to elucidate the regulatory role of Notch signaling in aging and OE regeneration.

## Materials and Methods

### Animals

Lgr5-EGFP-IRES-CreERT2 (#008875, harboring a “knock-in” allele that abolishes Lgr5 gene function and expresses EGFP and CreERT2 fusion protein from the Lgr5 promoter/enhancer elements), Rosa26-TdTomato (#007914, a Cre reporter strain with a loxP-flanked STOP cassette prevents transcription of the downstream red fluorescent protein), and RosaNICD (#008159, containing a sequence encoding an intracellular portion of the mouse Notch1 gene with a loxP-flanked STOP cassette, leading to elevating intracellular signal transduction and mimicking Notch signaling pathway activation by Cre reporter) mice were purchased from the Jackson Laboratory. Mice were housed with open access to food and water at the Experimental Animal Center of Eye, Ear, Nose, and Throat Hospital, Fudan University (Shanghai, China). The animals were housed in a temperature-controlled environment (23 ± 2°C) with a light/dark cycle of 12 h. All experiments were approved by the Shanghai Medical Experimental Animal Administrative Committee (Permit Number: 2009-0082). Both male and female mice were used in this study, and the data were grouped together because no sex difference was evident. All animals were maintained at C57BL/6J background.

### Chemical Treatment

All the reagents were purchased from Sigma Aldrich unless specified. The final concentration of chemicals used in organoid culture was: 5 μM LY411575 (#SML0506) and 40 ng/ml Jagged 1 (aa 1-1067, #SRP8012). LY411575 was dissolved in sterile-filtered DMSO (#D2650) while Jagged 1 was dissolved in sterile phosphate-buffered saline (PBS) to make stock solution. Chemicals were diluted by 1,000 times into the cultures. Organoids were treated with chemicals on Day 3 after passaging and incubated with chemicals for 4 days.

### Establishment of OE Injury Model

Methimazole leads to a dose-dependent olfactory toxicity (Genter et al., 1995). For OE lesion, animals were intraperitoneally injected with methimazole (50 μg/g body weight, #46429; Leung et al., 2007). In the saline control, the animals were injected with the same amount of PBS. The Lgr5-EGFP-CreERT2 mice were killed at Days 3, 5, 10, 17, and 32 after methimazole administration. Lgr5-EGFP-CreERT2/Rosa26-TdTomato (LT) and Lgr5-EGFP-CreERT2/Rosa26-TdTomato/RosaNICD (LTN) mice were killed at Day 30 postinjury for further analysis.

### Lineage Tracing and Notch Activation Mimicking

For lineage tracing and Notch activation mimicking in injured OE, 220 μg/g (body weight) tamoxifen (#T5648) was injected subcutaneously into LT and LTN mice for respective three consecutive days just prior to and after methimazole administration (the scheme is shown as Figure 6A). In the control group, mice were received with same doses of sunflower seed oil (#S5007), served as a solvent to tamoxifen. For lineage tracing in LT mice with different ages including 1-, 3-, 12-, and 24-month old, three doses of tamoxifen (1 dose/day) was injected prior to the methimazole treatment. The scheme for lineage tracing was shown as Figure 5A. For lineage tracing, administration of tamoxifen induced the generation of Cre, removed the STOP cassette, and induced the expression of TdTomato. Thus, all TdTomato+ cells were Lgr5+ cells or progenies derived from Lgr5+ cells. For Notch activation mimicking, Cre induced by tamoxifen administration led to the expression of NICD and then activated Notch signaling pathway.

**Figure 1:**
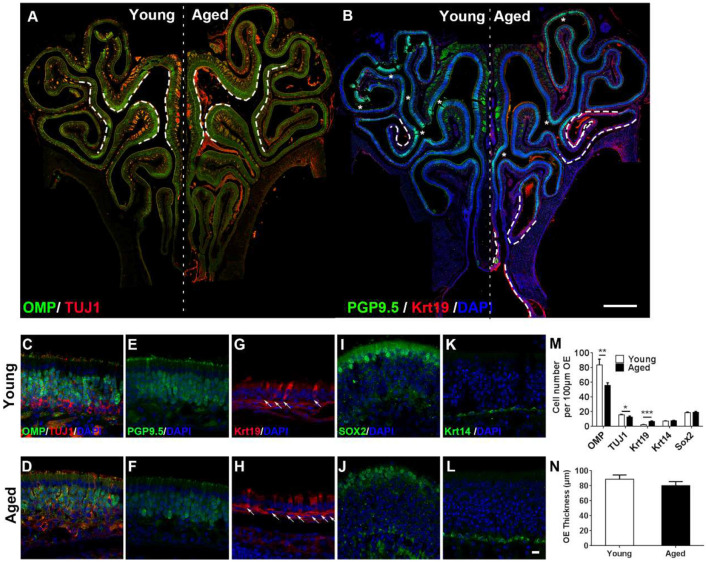
Aging altered neuronal generation in the olfactory epithelium (OE). **(A)** Immunostaining against mature and immature sensory neuronal markers olfactory marker protein (OMP) and TUJ1 in the OE of young and aged mice at 3- and 25-month old. The positive signals against OMP were labeled by dash lines. **(B)** Immunostaining against sensory neuronal marker PGP9.5 (noted by asterisks) and columnar-ciliated respiratory epithelial cell marker Krt19 (noted by dash lines). **(C–L)** Confocal images of staining against OMP and TUJ1 **(C,D)**, PGP9.5 **(E,F)**, Krt19 (**G,H**, for respiratory epithelial cells, labeled by arrows), Sox2 (**I,J**, for apical supporting cells), and Krt14 (**K,L**, for HBCs) in the OE of young and aged mice. Nuclei were counterstained with DAPI, as shown in blue. **(M)** Statistical analysis of OMP+, TUJ1+, Krt19+, Krt14+, and apical Sox2+ cell density in the OE of young and aged mice. In young and aged OE, 1,406 and 723 OMP+, 262 and 179 TUJ1+, 162 and 133 Krt19+, 98 and 139 Krt14+, and 182 and 222 apical Sox2+ cells were counted. **(N)** Statistical analysis of OE thickness from young and aged mice. Each OE thickness was calculated from 18 sections of three mice. The statistical significance was determined by unpaired *t*-test with Mann–Whitney correction. **p = 0.0072, ***p < 0.001 in **(M)**, *p = 0.3151 in **(N)**. Scale bars: 0.5 mm in **(B)** and 10 μm in **(L)**.

**Figure 2:**
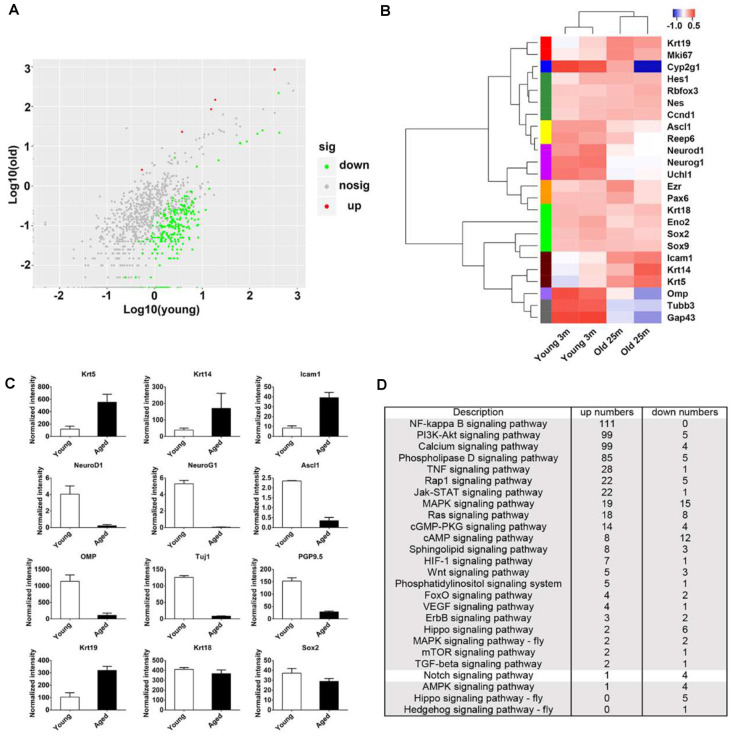
Transcriptional analysis on the OE from young and aged mice at 3- and 25-month old. **(A)** Scatter plot showing the up- and downregulated as well as nondifferentially expressed genes in the OE from young and aged mice. **(B)** Heatmap showed the significantly differentially expressed biomarkers for cellular lineages in the OE from young and aged mice. **(C)** Statistical analysis of differential mRNA levels of OE biomarkers using RNA-seq data. **(D)** KEGG enrichment analysis indicated differentially expressed genes between the OE from young and aged mice participating in multiple signaling pathways including Notch.

**Figure 3:**
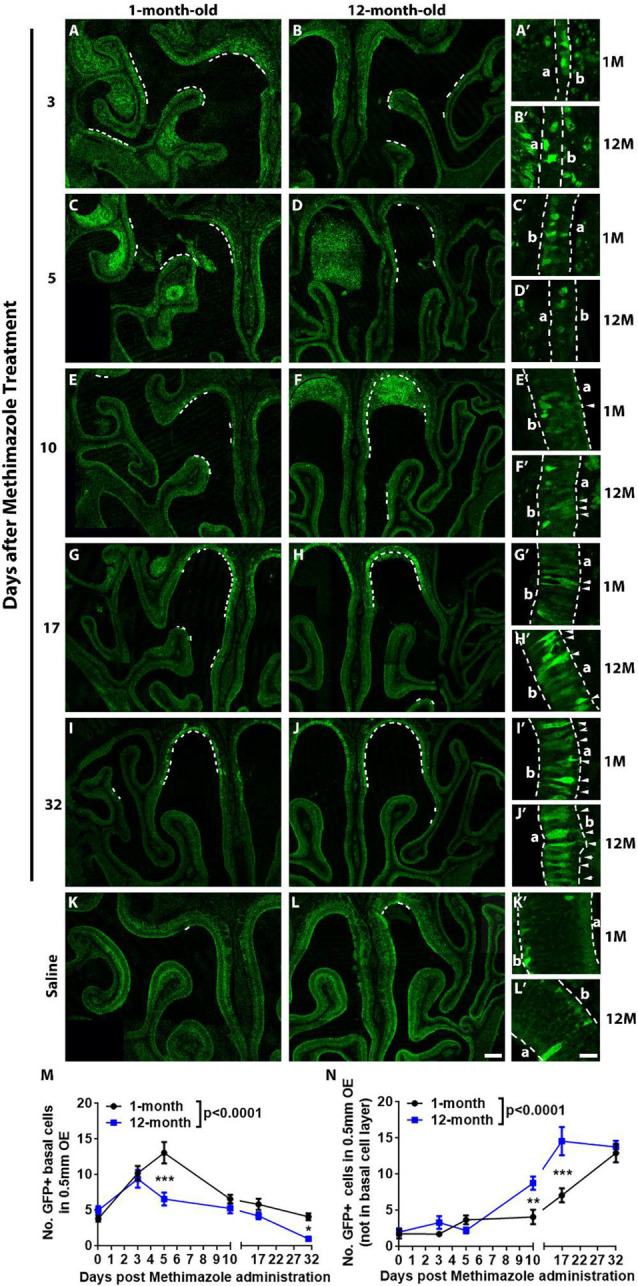
Aging led to the attenuation in recruitment of Lgr5+ cells in injured OE. Confocal images of Lgr5-EGFP+ cells in the OE of 1- and 12-month-old Lgr5-EGFP-CreERT2 mice at Day 3 **(A,B)**, Day 5 **(C,D)**, Day 10 **(E,F)**, Day 17 **(G,H)**, and Day 32 **(I,J)** postinjury as well as in the OE of uninjured saline control **(K,L)**. Positive staining against GFP was labeled by dash lines in **(A–L)**. Panels **(A’–L’)** were magnified regions in **(A–L)**. Lgr5-EGFP+ cells in the nonbasal layers were labeled by arrowheads. Dash lines in **(A’–L’)** represented boundary of the OE. **(M,N)** Statistical analysis of Lgr5-EGFP+ cell density in basal and nonbasal cell layers of injured OE from young and aged mice. At Days 3, 5, 10, 17, and 32 postinjury, 207, 798, 549, 336, 333, and 222 Lgr5-EGFP+ basal cells were counted in saline controls and in 1-month-old mice. In 12-month-old mice, 150, 480, 237, 348, 279, and 51 Lgr5-EGFP+ basal cells were counted. In 1- and 12-month-old mice, 111, 141, 144, 258, 447, 738 Lgr5-EGFP+ nonbasal cells and 66, 180, 87, 474, 831, and 744 Lgr5-EGFP+ nonbasal cells were counted. In **(A’–L’)**, **a** and **b** denote apex and base. The statistical significance was determined by two-way ANOVA. *F*_(5,200)_ = 24.75, *p* < 0.0001 between 1- and 12-month-old mice in **(M)**; *F*_(5,207)_ = 44.29, *p* < 0.0001 in **(N)**. The significance at the same-day postinjury between 1- and 12-month-old mice (noted by asterisks) was determined by Sidak’s multiple comparisons test. The significance at different time points postinjury compared with uninjured control was determined by unpaired *t*-test. Scale bars: 0.2 mm in **(L)** and 20 μm in **(L’)**.

**Figure 4:**
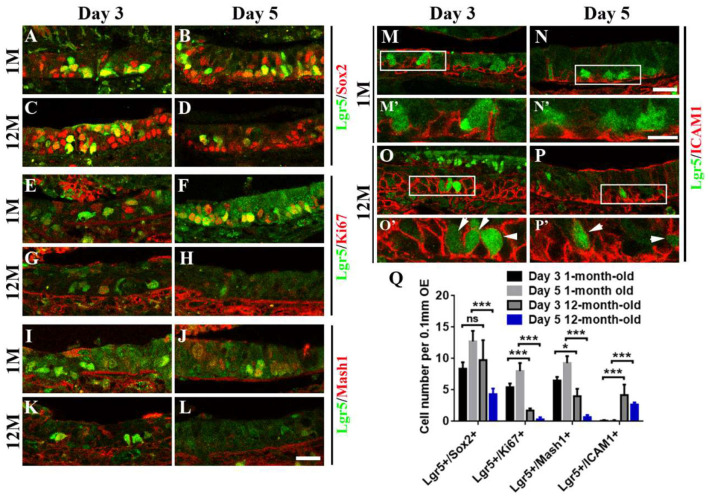
Aging altered the stemness of Lgr5+ cells in injured OE. Immunostaining against GFP and basal cell marker Sox2 **(A–D)**, GFP and proliferative cell marker Ki67 **(E–H)**, GFP and GBC marker Mash1 **(I–L)**, GFP and HBC marker ICAM1 **(M–P)** in the OE of 1- and 12-month-old Lgr5-EGFP-CreERT2 mice at Days 3 and 5 postinjury. Panels **(M’–P’)** were the magnification of squared regions in **(M–P)**. Lgr5-EGFP+/ICAM1+ cells were labeled by arrowheads in **(O’,P’)**. **(Q)** Statistical analysis on the density of Lgr5+/Sox2+, Lgr5+/Ki67+, Lgr5+/Mash1+, and Lgr5+/ICAM1+ cells in the OE of 1- and 12-month-old mice at Days 3 and 5 postinjury. At Days 3 and 5 postinjury, 603 and 420 Lgr5+/Sox2+ cells, 147 and 336 Lgr5+/Ki67+ cells, and 156 and 390 Lgr5+/Mash1+ cells were counted in 1-month-old mice. At Days 3 and 5, 375 and 195 Lgr5+/Sox2+ cells, 57 and 19 Lgr5+/Ki67+ cells, 108 and 18 Lgr5+/Mash1+ cells, and 153 and 72 Lgr5+/ICAM1+ cells were counted in 12-month-old mice. The statistical significance was determined by two-way ANOVA. *F*_(3,108)_ = 84.92, *p* < 0.0001. The asterisks were determined by Tukey’s multiple comparisons test. Scale bars: 20 μm in **(L,N)** and 10 μm in **(N’)**; ns, not significant.

**Figure 5:**
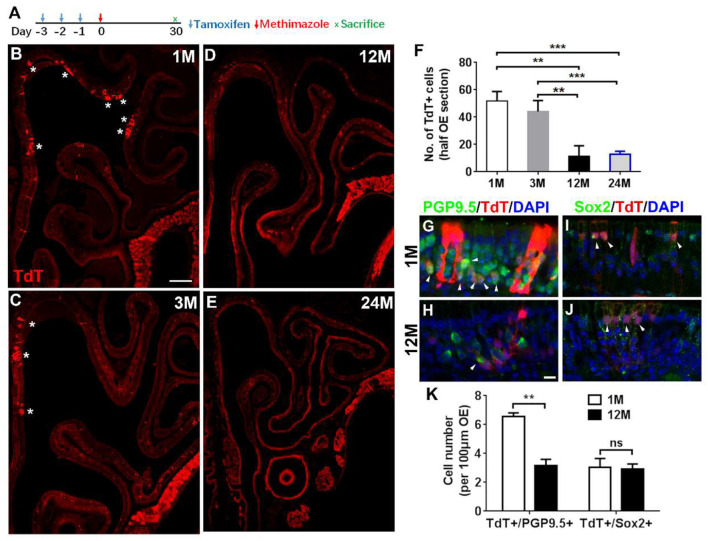
Aging reduced the progenies of Lgr5+ cells in injured OE. **(A)** Scheme showing the lineage tracing of Lgr5+ cells in Lgr5-CreERT2/Rosa26-TdTomato (LT) mice. **(B–E)** Confocal images of lineage-traced TdT+ cells in the OE of LT mice at 1-, 3-, 12-, and 24-month old at Day 30 postinjury. *In **(B)** and **(C)** denoted TdT+ cell bundles. **(F)** Statistical analysis of TdT+ cell density in the OE at Day 30 postinjury. Totally, 2, 525, 921, 766, and 289 TdT+ cells were counted in mice at 1-, 3-, 12-, and 24-month old (*n* = 3). **(G,H)** Immunostaining against PGP9.5 in the OE of 1- and 12-month-old LT mice at Day 30. TdT+/PGP9.5+ cells were noted by arrowheads in **(G,H)**. **(I,J)** Confocal images of TdT+/Sox2+ cells (arrowheads labeled) in the OE of 1- and 12-month-old LT mice at Day 30. **(K)** Statistical analysis on the density of TdT+/PGP9.5+ and TdT+/Sox2+ cells in LT mice at 1- and 12-month old. Statistical significance was determined by unpaired *t*-test. ns, not significant. In **(F)** and **(K)**, **p < 0.01, ***p < 0.001. Scale bars: 0.2 mm in **(B)** and 20 μm in **(H)**.

**Figure 6:**
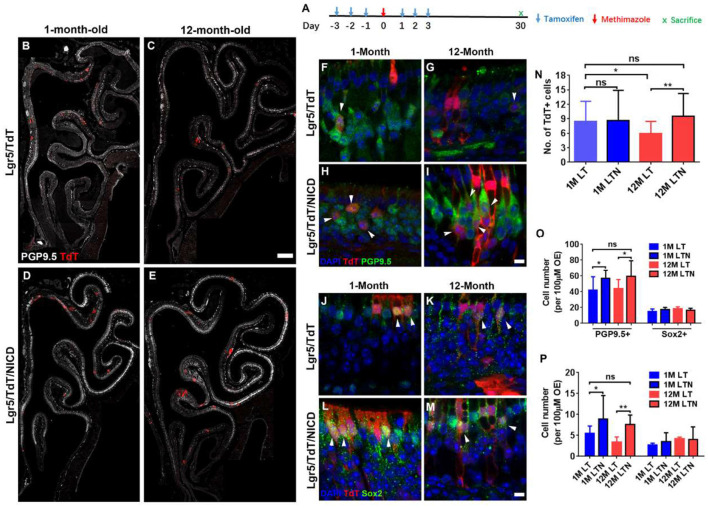
Notch activation recovered neuronal regeneration of Lgr5+ cells in aged OE. **(A)** Scheme showing the tamoxifen and methimazole administration in Lgr5/TdT (LT) and Lgr5/TdT/NICD (LTN) mice. **(B–E)** Images showing PGP9.5+ and TdT+ cells in the OE from LT and LTN mice at Day 30 postinjury. **(F–I)** Immunostaining against PGP9.5 in injured OE from LT and LNT mice at 1- and 12-month old. The TdT+/PGP9.5+ cells were labeled by arrowheads. **(J–M)** Confocal images of TdT+/Sox2+ cells (noted by arrowheads) in the OE from LT and LNT mice at Day 30 postinjury. **(N)** Statistical analysis of TdT+ cell density (cell number per 100 μm OE) in LT and LTN mice at 1- and 12-month old. Totally, 461, 503, 421, and 588 TdT+ cells were counted in young LT, young LTN, aged LT, and aged LTN mice. **(O)** Statistical analysis of PGP9.5+ and Sox2+ cell density in the OE from young and aged LT and LNT mice at Day 30 postinjury. 1,256, 1,716, 1,419, and 1,429 PGP9.5+ cells and 224, 311, 329, and 247 Sox2+ cells were counted in young LT, young LTN, aged LT, and aged LTN mice. **(P)** Statistical analysis of TdT+/PGP9.5+ and TdT+/Sox2+ cell density in the OE. In young LT, young LTN, aged LT, and aged LTN mice, 103, 207, 125, and 172 TdT+/PGP9.5+ cells and 42, 62, 75, and 81 TdT+/Sox2+ cells were counted. The statistical significance was determined by unpaired *t*-test in **(N)** and by two-way ANOVA with Sidak’s multiple comparisons test in **(O)** and **(P)**. ns, not significant. *p < 0.05, **p < 0.01.

### Immunofluorescence Staining

Mice were deeply anesthetized with an intraperitoneal injection of sodium pentobarbital (40 mg/kg, #P3761) and perfused transcardially with 0.9% saline, followed by 4% paraformaldehyde (#16005). Brains were postfixed overnight in the 4% paraformaldehyde and then were decalcified in 0.5 M EDTA (#15575020, Invitrogen™) until the skull became transparent. After being washed in PBS, tissues were equilibrated sequentially in 10, 20, and 30% sucrose (#S0389) and embedded in Tissue-Tek O.C.T. Compound (#4583, Sakura). OE tissues were cryosectioned to 20 μm slices using a Leica CM3050S Cryostat.

Immunofluorescence staining was performed according to a standard protocol. After rinsing with PBST (PBS plus 0.3% Triton X-100), the sections were blocked in PBST with 5% bovine serum albumin (#0332, AMRESCO) for 60 min, and then incubated with the primary antibodies overnight at 4°C. Then, the sections were incubated in secondary antibodies at room temperature for 1 h. The nuclei were counterstained with DAPI (#D3571, Thermo Fisher Scientific). Tissues were mounted in Vectashield Mounting Medium (#H1200, Vector Laboratories). Images were captured under a confocal laser scanning microscope ZEISS with Zen Software. The primary antibodies used in the immunostaining and their marked cellular subtypes, dilutions, vendors, and category numbers were as follows: rabbit anti-OMP (#ab87338, Abcam, 1:200, mature olfactory sensory neurons), mouse anti-TUJ1 (#ab78078, Abcan, 1:200, immature olfactory sensory neurons), rabbit anti-PGP9.5 (#14730-1-AP, Proteintech, 1:400, olfactory sensory neurons), mouse anti-Krt14 (#10143-1-AP, Proteintech, 1:200, horizontal basal cells), mouse anti-Krt19 (TROMA-III-c, DSHB, 1:300, ciliated respiratory epithelial cells), rabbit anti-Sox2 (#ab92494, Abcam, 1:200, supporting and basal cells), chicken anti-GFP (#ab13970, Abcam, 1:1,000, Lgr5-EGFP+ cells), rabbit anti-GFP (#A11122, Thermo Fisher Scientific, 1:500, Lgr5-EGFP+ cells), goat anti-Sox2 (#sc-17320, Santa Cruz Biotechnology, 1:200, supporting and basal cells), mouse anti-Ki67 (#550609, BD Biosciences, 1:100, proliferative basal cells), goat anti-ICAM1(#AF796, R&D, 1:500, horizontal basal cells), and mouse anti-Mash1 (#556604, BD Biosciences, 1:100, globose basal cells). All the secondary antibodies used in this study were purchased from Thermo Fisher Scientific and diluted as 1:300, including Alexa Fluor 594 Donkey anti-Rabbit (#A21207), Alexa Fluor 488 Donkey anti-Rabbit (#A21206), Alexa Fluor 594 Donkey anti-Goat (#A11058), Alexa Fluor 568 Donkey anti-Mouse (#A10037), Alexa Fluor 488 Donkey anti-Goat (#A11055), Alexa Fluor 633 Donkey anti-Goat (#A21082), Alexa Fluor 488 Donkey anti-Mouse (#A21202), Alexa Fluor 647 Donkey anti-Mouse IgG (#A31571), and Alexa Fluor 647 Donkey anti-Rabbit (#A31573).

### OE Organoid Culture

Single cells from OE were cultured according to previously reported protocol with some modifications (Dai et al., 2018). Briefly, intact nasal mucosa of C57BL/6J mice at 3- and 19-month old was dissected and minced into small pieces using scissors in Tyrode’s solution (145 mM NaCl, 5 mM KCl, 10 mM HEPES, 5 mM NaHCO3, 10 mM pyruvate, 10 mM glucose). Tissues were digested in 0.25% Trypsin-EDTA (#25200056, Gibco™) and 10 mg/ml DNase I (#11284932001, Roche) for 20 min at 37°C. Then samples were centrifuged at 1,200 r.p.m. for 5 min, and supernatant was discarded; ~1 ml organoid growth medium (see below, no addition of Matrigel) was added, and tissues were mechanically dissociated into single cells using a 1-ml fire-polished syringe needle. Single cell suspension was filtered with 40-μm nylon mesh cell strainers (#352235, BD Falcon). Cells were seeded in ultra-low-attached 24-well plates (#3473, Corning) at density of ~1 × 10^4^ cells per well. OE organoid growth medium was based on DMEM/F12 medium (#10565018, Thermo Fisher Scientific) supplemented with R-Spondin-1 (200 ng/ml, #4645-RS, R&D), Noggin (100 ng/ml, #250–38, PeproTech), Wnt3a (50 ng/ml, #5036-WN-010, R&D), Y27632 (10 μM, #Y0503, Sigma Aldrich), epidermal growth factor (EGF, 50 ng/ml, #PHG0311, Thermo Fisher Scientific), N2 (1%, #17502001, Thermo Fisher Scientific), B27 (2%, #17504044, Thermo Fisher Scientific), HEPES (1 mM, #15630080, Thermo Fisher Scientific), and Matrigel (3% (Vol/Vol), #356231, BD Biosciences). Medium was changed every 3 days. Visible organoids were observed at Day 3 after seeding. For passaging, organoids at Day 10 after *in vitro* culture were digested using 0.25% Trypsin-EDTA for 10 min at 37°C. Organoids were resuspended in culture medium without addition of Matrigel, and single cell suspension was prepared by drawing through a 1-ml microsyringe. Cells were reseeded in ultra-low-attached 24-well plates at density of ~5,000 cells per well.

### RNA Sequence

The protocol for RNA sequence was described previously (Ren et al., 2020). RNA was prepared from mouse olfactory mucosa using TRIZOL reagent. RNA-Seq analysis was performed by Majorbio Corp. (Shanghai, China). Briefly, sequencing reads were mapped to the mouse genome using HISAT2. Transcriptome from RNA-seq reads was reconstructed by StringTie, and expression differences were evaluated using DESeq2. Pearson’s coefficient was calculated to determine the correlation among different groups. The clustering analysis of the global gene expression pattern in different samples was carried out using K-means clustering algorithm by RSEM software. Gene Ontology (GO) and Kyoto Encyclopedia of Genes and Genomes (KEGG) pathway analysis were performed at http://www.geneontology.org/ and http://www.genome.jp/kegg/. I-Sanger (www.i-sanger.com) was used to analyze all the sequence data.

### Statistical Analysis

Each experiment was repeated in triplicate independently. Quantitative data were expressed as mean ± SEM. The evaluators involved in assessing organoid size and cell counting were blinded to the experimental design including age and genotypes of the animals from which the specimens were derived. Organoid counting for the ratio of cystic and filled appearance were based on the images captured under bright field. The number of positively stained cells (including OMP+, Tuj1+, Krt19+, Krt14+, Sox2+, Lgr5-EGFP+, Lgr5+/Sox2+, Lgr5+/Ki67+, Lgr5+/Mash1+, Lgr5+/ICAM1+, TdTomato+, PGP9.5+, TdTomato+ (TdT+)/PGP9.5+, and TdT+/Sox2+ cells), organoid size, and OE thickness were measured by Image J Software. All the cell counting data were derived from the number of cells on six OE sections per mice. We biologically triplicated each experiment, thus 18 sections from three animals were used for each cell counting analysis. Each slide had OE sections from anterior to posterior zones. On each OE section, six different regions (three in dorsal and three in ventral) were selected to measure the cell density. These selected regions were consistent among all groups. The density of positively stained cells was defined as the number of positively stained cells per 100 μm or 0.5 mm OE. The density of TdT+ cells in Figure 5 was defined as the number of TdT+ cells per OE section. The OE thickness was averaged from five different places on each OE section. Two-way ANOVA with Sidak’s multiple comparisons test and Tukey’s multiple comparisons test was used to determine the statistical difference among multiple groups, while comparison between two groups was performed by unpaired *t*-test using GraphPad Prism 6.0 software. *p* values < 0.05 were considered statistically significant.

## Results

### Aging Attenuates Sensory Neuronal Generation in the OE

To elucidate the effect of aging on the OE, we performed immunostaining against various biomarkers for cellular subtypes constituting the OE in wild-type C57BL/6J mice at 3- and 25-month old. The intensity of staining against mature sensory neuronal markers OMP was weaker in the OE of aged animals compared with the young mice (Figures 1A,C,D, differential OMP+ staining signals were noted by dash lines in Figure 1A). The density of OMP+ cells was decreased by 28.0 ± 4.4% in aged OE compared with the young one (Figure 1M, *p* = 0.0072, *n* = 3 mice). The intensity of staining against immature neuronal marker TUJ1 did not show apparent difference (Figures 1A,C,D), while the density of TUJ1+ cells in aged OE was still significantly lowered by 18.3 ± 4.9% than the OE of young mice (Figure 1M, *p* = 0.0213, *n* = 3 mice). Besides, the intensity of PGP9.5+ staining signals in the OE of young mice was more intensive than the old animals (Figures 1B,E,F, PGP9.5+ staining signals were labeled by asterisks in Figure 1B). By contrast, staining against Krt19, a biomarker for columnar ciliated respiratory epithelial cells, was more obvious in the OE of aged animals compared with the young mice (Figure 1B, noted by dash lines, Figures 1G,H, noted by arrows). The density of Krt19+ cells in the OE from aged mice was significantly increased by 2.3 ± 0.3-fold, compared with the young OE (Figure 1M, *p* < 0.001). However, we did not observe the apparent change in apical Sox2+ staining (marker for sustentacular cells, Figures 1I,J) or in Krt14+ staining (marker for HBCs, Figures 1K,L). The density of apical Sox2+ sustentacular cells or Krt14+ basal cells was not significantly changed in the OE between aged and young animals (Figure 1M, *p* = 0.3769 for Krt14+ cells and *p* = 0.6724 for apical Sox2+ cells, *n* = 3). The OE thickness of aged mice was not significantly different from the young animals (Figure 1N, *p* = 0.3151). Thus, we concluded that aging led to the attenuation in mature sensory neuronal generation in the OE.

### Transcriptional Alteration in Aged OE

To elucidate the panoramic effect of aging on the OE, we performed transcriptional analysis between the OE from aged and young animals at 3- and 25-month old, respectively. Totally, 1,342 upregulated and 1,005 downregulated genes were found in aged OE. By KEGG enrichment analysis, we found five upregulated and 265 downregulated genes associated with olfactory transduction in the OE of aged mice (Figure 2A). The heatmap showed that the expression level of HBC markers Krt14, Krt5, ICAM1, and respiratory epithelial cell marker Krt19 was increased in aged OE, while level of GBC markers NeuroD1, NeuroG1, Ascl1, neuronal marker OMP, Tuj1, and GAP43, as well as sustentacular cell marker Cyp2g1 was decreased (Figures 2B,C). KEGG pathway analysis found that genes involved in inflammation pathway such as NF-kappa B, PI3K-Akt, TNFα, JAK/STAT, and MAPK signaling were upregulated in aged OE (Figure 2D). Genes participating in critical signal pathways such as Wnt, Notch, and Hippo were differentially expressed between the OE from young and aged mice (Figure 2D). Thus, we provided a transcriptional landscape showing the alteration in the OE with aging.

### Aging Leads to the Attenuation in Recruitment of Lgr5+ Cells in Injured OE

Lgr5 marks GBCs and Lgr5+ GBCs are recruited in injured OE (Chen et al., 2014; Dai et al., 2018). Since GBC markers such as Ascl1, NeuroD1, and NeuroG1 at transcriptional level were decreased in aged OE (Figures 2B,C), we determined whether aging weakened the recruitment of Lgr5+ GBCs in lesioned OE. Lgr5-EGFP-CreERT2 mice at 1- and 12-month old were injected with methimazole, and the density of Lgr5-EGFP+ cells was measured postlesion. The recruitment of Lgr5-EGFP+ cells in injured OE was not significantly different at Day 3 postinjury between 1- and 12-month-old mice (Figures 3A,B) but significantly retarded at Day 5 in aged OE (Figures 3C,D). Statistically, the density of Lgr5-EGFP+ cells was significantly increased in injured OE of both 1- and 12-month-old mice at Day 3 postlesion compared with the unlesioned controls (Figure 3M, *p* < 0.001 by unpaired *t*-test, *n* = 3). However, the density of Lgr5-EGFP+ cells was still drastically enhanced at Day 5 in young animals (*p* < 0.001 compared with Day 3, *n* = 3) while the density was reduced in the OE of 12-month-old mice at Day 5 compared with the density at Day 3 postinjury and not significantly changed in contrast to the saline control (Figure 3M). At Days 10, 17, and 32 postinjury, the density of Lgr5-EGFP+ cells at basal cell layer in the OE of both 1- and 12-month-old mice continued to decrease in contrast to the density at Day 5 and returned to the threshold level similar as the density of Lgr5-EGFP+ cells in the saline control (Figures 3E–M). Compared with the OE of young mice, the density of Lgr5-EGFP+ cells in aged OE at Days 5 and 32 postinjury was significantly decreased (Figure 3M, *p* < 0.001 at Day 5 and *p* < 0.05 at Day 32 using two-way ANOVA with Sidak’s multiple comparisons test). Surprisingly, the Lgr5-EGFP+ cells appeared in the nonbasal cell layer during the recovery of OE from methimazole-induced lesion at Days 10, 17, and 31 (Figures 3E–J, labeled by arrowheads in Figures 3E′–J′). The density of nonbasal Lgr5-EGFP+ cells was significantly increased at Days 10, 17, and 31 postinjury in the OE of 12-month-old mice, compared with the density in unlesioned controls (Figure 3N, *p* < 0.001 by unpaired *t*-test, *n* = 3), while the density of Lgr5+ nonbasal cells was dramatically increased at Days 17 and 31 postinjury in the OE of young animals (Figure 3N, *p* < 0.001, *n* = 3). Compared with the young mice, the density of nonbasal Lgr5-EGFP+ cells in the aged OE was significantly increased at Days 10 and 17 postinjury (Figure 3N, *p* < 0.01 at Day 10 and *p* < 0.001 at Day 17 using two-way ANOVA with Sidak’s multiple comparisons test). Collectively, these data demonstrated that aging weakened the recruitment of Lgr5+ basal cells in injured OE.

### Aging Alters the Stemness of Lgr5+ Cells in Injured OE

Since aging attenuated the recruitment of Lgr5+ basal cells in injured OE, we determined whether the stemness of Lgr5+ cells was affected in aged mice compared with the young ones. At Days 3 and 5 postinjury, we observed the apparent recruitment of Lgr5+/Sox2+ basal cells (Figures 4A,B), Lgr5+/Ki67+ proliferative cells (Figures 4E,F), and Lgr5+/Mash1+ GBCs (Figures 4I,J) in the OE of 1-month-old mice. By contrast, Lgr5+/Ki67+ and Lgr5+/Mash1+ cells were only recruited at Day 3 in injured OE of 12-month-old mice, but their densities was significantly decreased compared with the counterparts in young mice at Day 3 (Figures 4E,G,I,K,Q, *p* < 0.001 for Lgr5+/Ki67+ cell density, *p* < 0.05 for Lgr5+/Mash1+ cell density). However, there was barely Lgr5+/Ki67+ or Lgr5+/Mash1+ cells in injured OE of aged mice at Day 5 (Figures 4H,L,Q). However, Lgr5+/Sox2+ cells were apparently activated at Day 3 and still present at Day 5 postinjury in the OE of 12-month-old mice (Figures 4C,D). In 1-month-old mice, Lgr5+ cells were not co-immunostained with ICAM1, a biomarker for HBCs, at Days 3 and 5 postlesion (Figures 4M,N, squares were magnified as Figures 4M′,N′). By comparison, Lgr5+/ICAM1+ cells were found in the OE of 12-month-old mice at Days 3 and 5 postinjury (Figures 4O,P, labeled by arrowheads in magnified squares as Figures 4O′,P′). The density of Lgr5+/ICAM1+ cells was significantly increased in injured OE of 12-month-old mice at Days 3 and 5, compared with the density in the OE of 1-month-old mice (Figure 4Q, *p* < 0.001 by two-way ANOVA with Tukey’s multiple comparisons test). This showed a portion of Lgr5+ cells remained in dormancy in injured OE of aged mice. Collectively, we concluded that aging led to the alteration in stemness of injury-recruited Lgr5+ cells in the OE, keeping some Lgr5+ cells in dormancy after OE injury.

### Aging Weakens Progeny Generation From Lgr5+ Cells in Injured OE

Since Lgr5+ cells in the OE from young and aged animals were differentially recruited postinjury, we then determined whether the progeny generation from Lgr5+ cells in injured OE was affected by aging. Lineage tracing of Lgr5+ cells in LT mice through tamoxifen administration (Figure 5A) showed the TdT+ cell bundles (progenies from Lgr5+ cells) were obvious in the OE from mice at 1- and 3-month old (Figures 5B,C, noted by asterisks). In mice at 12-month old, single TdT+ cells were scattered throughout the OE while the cell bundles were seldom observed (Figure 5D). However, fewer TdT+ cells were present in injured OE of LT mice at 24-month old (Figure 5E). Statistical analysis indicated that the density of TdT+ cells (number of TdT+ cells per half OE section) was significantly lowered by 77.7 ± 14.4 and 75.3 ± 4.4% in injured OE of 12- and 24-month-old mice compared with that in the OE of 1-month-old animals (Figure 5F, *p* = 0.0014 and *p* = 0.0002 by unpaired *t*-test). Similarly, the density of TdT+ cells in the OE of 12- and 24-month-old mice was decreased by 73.8 ± 16.9 and 70.9 ± 5.1% compared with the density in mice at 3-month old (Figure 5F, *p* = 0.0055 and *p* = 0.0002). Furthermore, we observed the apparent TdT+/PGP9.5+ cells in the OE of young mice at Day 30 postinjury (Figure 5G, noted by arrowheads), while significantly fewer TdT+/PGP9.5+ cells were found in the OE of 12-month-old mice at Day 30 postinjury (Figure 5H). Statistically, the density of TdT+/PGP9.5+ cells in LT mice at 12-month old was decreased by 51.6 ± 6.6% compared with 1-month-old mice (Figure 5K, *p* = 0.0025). Meanwhile, TdT+/Sox2+ cells were present in the OE of both 1- and 12-month old at Day 30 and did not show the apparent difference (Figures 5I–K, *p* = 0.8356, noted by arrowheads). Therefore, aging dampened neuronal differentiation of Lgr5+ cells in injured OE.

### Notch Activation Strengthens Neuronal Regeneration in Aged OE

To determine whether the Notch signaling pathway could differentially regulate the neuronal regeneration in injured OE of young and aged mice, LTN mice at 1- and 12-month old were injected with tamoxifen to induce the overexpression of NICD (Figure 6A). With Notch activation mimicking, we found the significant increase in TdT+ cell density by 31.8 ± 6.5% in the OE of 12-month-old LTN mice compared with the density in 12-month-old LT mice (Figures 6C,E,N, *p* = 0.0081 by unpaired *t*-test). However, the TdT+ cell density was not significantly different between the OE of 1-month-old LT and LTN mice (Figures 6B,D,N, *p* = 0.8864). Furthermore, the TdT+ cell density in the OE of LTN mice at 12-month old was comparable with that in the OE of LT mice at 1-month old (Figure 6N, *p* = 0.4518), suggesting Notch activation enhanced the progeny generation from Lgr5+ cells in the OE of aged mice to the similar level as in the young animals. Staining against PGP9.5 was denser in the OE of LTN mice at both 1- and 12-month old, compared with the counterparts in LT mice (Figures 6B–E). The density of PGP9.5+ cells was higher in LTN mice at both 1- and 12-month old by 36.8 ± 7.4 and 26.7 ± 5.9%, compared with the counterparts in LT mice (Figure 6O, *p* < 0.05 by two-way ANOVA with Sidak’s multiple comparisons test). By contrast, the density of apical Sox2+-supporting cells did not show a significant difference in the OE of LT and LTN mice at either 1- or 12-month old (Figure 6O, *p* > 0.1). The TdT+/PGP9.5+ cell density was significantly increased by 60.8 ± 14.5% (*p* < 0.05) and 125.3 ± 25.0% (*p* < 0.01) in the OE of young and aged LTN mice compared with the density in LT mice (Figures 6F–I,P, noted by arrowheads), while the density of TdT+/Sox2+ cells did not apparently change with Notch activation (Figures 6J–M,P, *p* > 0.1). Thus, Notch activation mimicking through NICD overexpression enhanced the neuronal regeneration capacity in aged OE postinjury, making it comparable with the young OE.

### Notch Regulates Aging-Induced Morphological Alteration in OE Organoids

**Figure 7:**
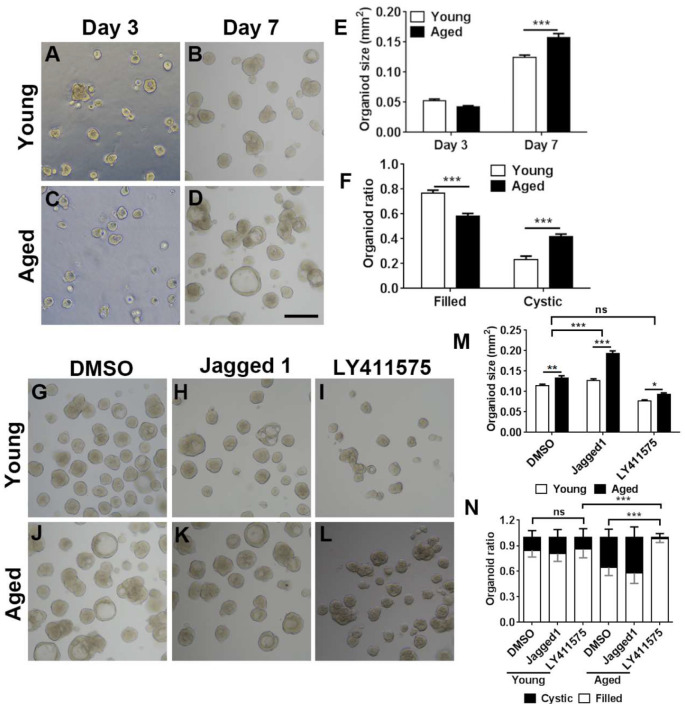
Chemical cocktail regulated organoid growth and morphology *via* Notch signaling pathway. **(A–D)** Images showing the morphological difference in organoids derived from the OE of young (3-month-old) and aged (19-month-old) mice at Days 3 and 7 after *in vitro* culture. **(E)** Statistical analysis on size of organoids from young and aged mice at Days 3 and 7. At Days 3 and 7, 391 and 501, 1,113, and 604 organoids from aged and young OE were measured. **(F)** Statistical analysis on the ratio of filled and cystic organoids from young and aged mice; 825 and 934 organoids from young and aged OE were counted. **(G–L)** Images showing organoids from young and aged animals in the presence of DMSO **(G,J)**, Notch activator Jagged 1 **(H,K)**, and Notch inhibitor LY411575 **(I,L)**. **(M)** Statistical analysis on size of organoids treated with DMSO, Jagged 1, or LY411575; 518 and 415, 463 and 505, and 359 and 379 organoids from young and aged OE were measured when treated with DMSO, Jagged 1, or LY411575. **(N)** Statistical analysis on ratio of cystic and filled organoids from young and aged mice, treated with DMSO, Jagged 1, or LY411575; 973 and 1, 040, 518 and 717, and 683 and 300 organoids from young and aged OE were counted in the presence of DMSO, Jagged 1, and LY411575. The statistical significance was determined by two-way ANOVA with Sidak’s multiple comparisons test. *F*_(1,814)_ = 4.533, *p* = 0.0335 in **(E)**; *F*_(1,56)_ = 209.9, *p* < 0.0001 in **(F)**; *F*_(2,1,493)_ = 147.3, *p* < 0.0001 in **(M)**. The statistical significance among different stimulation groups in **(M)** was determined by unpaired *t*-test. ns, not significant, *p < 0.05, **p < 0.01, ***p < 0.001. Scale bar: 250 μm.

We then determined whether Notch signaling could regulate the aging-induced variation in OE organoid growth *in vitro*. The organoid size was not significantly different at Day 3 postculture but drastically increased in organoids from aged mice (19-month-old) at Day 7 (Figures 7A–E, *p* < 0.001 by two-way ANOVA with Sidak’s multiple comparisons test). Compared with the organoids from young mice (3-month-old), percentage of organoids from aged mice with “filled” morphology was decreased by 23.7 ± 2.8%, while the ratio of cystic organoids was increased by 42.1 ± 2.8% (Figures 7A–D,F, *p* < 0.001). Then, we elucidated whether regulation of Notch signaling by small molecule chemicals *in vitro* could affect the organoid size and morphology. In the presence of Notch activator Jagged1, the size of organoids from aged mice was increased by 51.9 ± 5.1% compared with the organoids derived from young OE (Figures 7H,K,M, *p* < 0.001 by two-way ANOVA with Sidak’s multiple comparisons test). By contrast, size of DMSO-treated control organoids from young animals was only increased by 16.2 ± 4.7% (Figures 7G,J,M, *p* < 0.01). The size change in organoids from young and aged mice was significantly different between DMSO- and Jagged1-treated groups (*p* < 0.001 by unpaired *t*-test). Notch inhibitor LY411575 led to an increase in organoid size only by 21.7 ± 4.0% (Figures 7I,L,M, *p* < 0.05), which was not significantly different from the size change between DMSO-treated organoids from young and aged OE (*p* > 0.4). Furthermore, regulation of Notch signaling also changed the organoid morphology. LY411575 treatment led to significant increase in ratio of filled organoids from aged OE by 52.1 ± 6.5% compared with the DMSO-treated organoids (Figures 7J,L,N, *p* < 0.001). This was even higher than the ratio of filled organoids in untreated and LY411575-treated cultures from young mice by 16.0 ± 5.0 and 14.1 ± 4.9% (Figures 7G,I,L,N, *p* < 0.001). However, no apparent morphological change was observed in LY411575-treated organoids from young OE compared with the DMSO-treated control (Figure 7N). Collectively, these data demonstrated that regulation of Notch signaling by chemical stimulation affected aging-induced alteration in organoid size and morphology.

## Discussion

In this study, we reported the cellular and transcriptional alteration between young and aged OE. Aging weakened the recruitment of Lgr5+ cells, altered the stemness, and attenuated generation of neuronal progenies from Lgr5+ cells in the OE postinjury. Notch activation could recover the neuronal differentiation in aged OE, making it comparable with the OE of young animals. Therefore, this work provided the evidence to support the correlation between Notch signaling activation and alleviation in aging-induced incapacity of neuronal regeneration in injured OE, potentially providing a new concept in the therapy against aging-triggered anosmia.

Lgr5+ cells are active globose basal cells and are recruited in injured OE (Chen et al., 2014; Dai et al., 2018). However, it is still not clear whether dormant Lgr5+ cells are present in the OE, and this may lead to the discrepancy in the recruitment of Lgr5+ cells when OE is exposed to toxic reagent. Previous reports showed that Lgr5+ label-retaining cells were dormant stem cells in intestine (Li et al., 2016) and Lgr5+ intestinal stem cells were in G1 phase characterized by dormant periods (Carroll et al., 2018), showing the presence of Lgr5+ quiescent stem cells. In mammary gland, the deeply quiescent Lgr5+ mammary stem cells resided within the proximal region (Fu et al., 2017). These cells remained active in embryonic mammary primordia before switching to a quiescent state after birth and can re-enter the cell cycling under ovarian hormone stimulation (Fu et al., 2017). This resembles the situation of Lgr5+ cells in the OE, which were active in early postnatal stage, remained in dormancy in adults, and were activated by chemical lesion (Chen et al., 2014; Dai et al., 2018). In aged OE, the recruitment of Lgr5+ cells was attenuated compared with that in young OE (Figure 3). This raises the possibility that more Lgr5+ cells in aged OE postinjury are still in quiescence. This was supported by the observation that Lgr5+/ICAM1+ cells were present in aged OE at Days 3 and 5 postlesion while it was seldom found in young OE (Figure 4). Thus, driving the quiescent stem cell to enter the cell cycle is applicable to alleviate aging-induced smell loss.

Multiple signaling pathways (ON or OFF state of Wnt, BMP, or IP3K signaling) regulated transition between quiescent and active state of intestinal stem cells (Richmond et al., 2016). Through RNA-Seq analysis, we found the transcriptional alteration in several signaling pathways between the OE from young and aged mice (Figure 2). A few pathways have been reported in previous studies to determine the stemness of active rapid cycling and dormant stem cells, such as Wnt (Li and Clevers, 2010) and PI3K-Akt (Richmond et al., 2015). Meanwhile, Notch signaling was involved in multiple diseases associated with advanced aging (Sun et al., 2013; Rizzo et al., 2018). Here, we found that Notch activation mimicking by NICD overexpression could restore the neuronal differentiation of Lgr5+ cells in aged OE (Figure 6), while aging weakened capacity to generate progenies from Lgr5+ cells compared with the young OE (Figure 5). Members of the Notch signaling pathway were significantly downregulated in HBCs following chemical lesion, demonstrating Notch pathway participated in activation of HBCs postinjury (Herrick et al., 2017). Our previous work showed that Notch activation enhanced the differentiation of Lgr5+ cells in the normal OE (Dai et al., 2018). Based on these data, we further elucidated that Notch activation offset the impairment in neuronal differentiation of Lgr5+ cells in aged OE postinjury and made it comparable with the young OE (Figure 6). This supplemented the role of Notch signaling in recovering the aging-induced attenuation of neuronal regeneration in injured OE, besides maintaining the cell turnover in normal OE.

OE organoid is an ideal tool to study the cellular proliferation and differentiation, mimicking the process in the OE tissue (Dai et al., 2018; Chen et al., 2020). Organoids showed the various morphologies, but the significance of it was still not very clear. Previous reports indicated that human intestinal organoids underwent maturation with morphological alteration (Jung et al., 2018). Morphological variation in Lgr5+ embryonic liver cell-formed organoids represented different cellular subtypes (Prior et al., 2019). Thus, different morphologies might represent various extents in cellular proliferation and differentiation. The OE from young and old mice as well as humans contained differential density of OMP+ and Tuj1+ neurons (Figure 1; Child et al., 2018). The increasing ratio of cystic organoids derived from old animals potentially showed their limited differentiation capacity. Furthermore, our data showed that addition of LY411575 in organoids from aged OE increased the ratio of filled organoids (Figure 7N), suggesting Notch signaling regulated organoid morphology and potentially affected the cellular differentiation in organoids. This will be strengthened by using OE organoids from RosaNICD mice to elucidate whether Notch activation could offset the aging-induced morphological alteration and cellular differentiation *in vitro*. This needs to be further investigated at cellular and transcriptional levels.

In conclusion, this work showed the attenuation in Lgr5+ cell recruitment in aged OE. Generation of neuronal progenies from Lgr5+ cells in aged OE postinjury was recovered through Notch activation, making it comparable with the young OE. Thus, we provided new evidence to support the role of Notch in regulating aged OE regeneration, potentially putting forward new therapeutic concept against smell loss in elder people.

## Data Availability Statement

The raw data supporting the conclusions of this article will be made available by the authors, without undue reservation.

## Ethics Statement

The animal study was reviewed and approved by the Shanghai Medical Experimental Animal Administrative Committee (Permit Number: 2009-0082).

## Author Contributions

YY designed the research. YY, XL, MT, and LW performed the research. HY and YQ contributed unpublished reagents/analytic tools. YY and XL analyzed data and wrote the article. All authors edited the article. All authors agree to be accountable for the content of the work. All authors contributed to the article and approved the submitted version.

## Conflict of Interest

The authors declare that the research was conducted in the absence of any commercial or financial relationships that could be construed as a potential conflict of interest.
